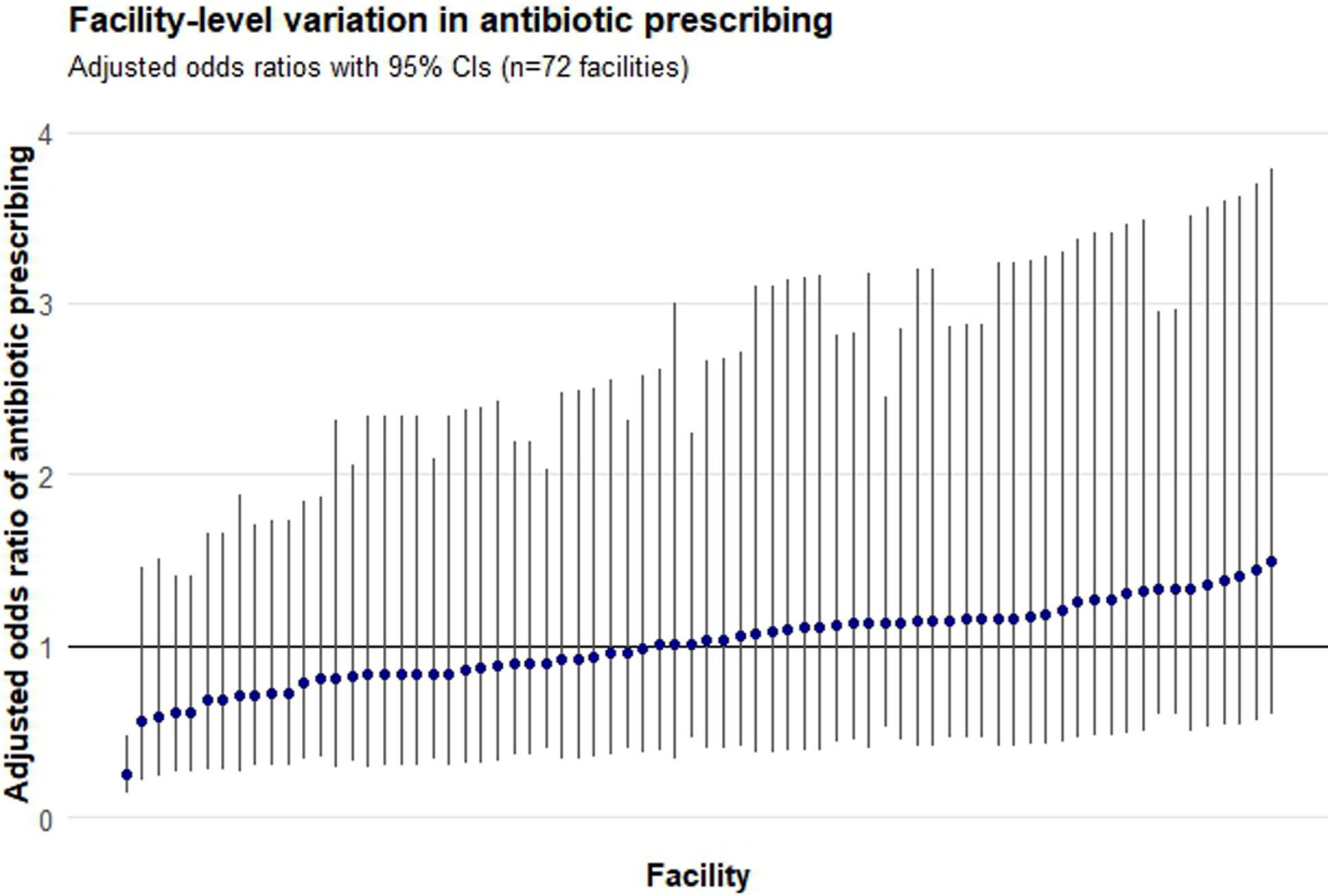# 61 How Low is Low Enough? Determining a Goal Central Line Standardized Utilization Ratio Necessary to Decrease Infections

**DOI:** 10.1017/ash.2026.10492

**Published:** 2026-06-23

**Authors:** Shinya Hasegawa, Hiroyuki Suzuki

**Affiliations:** 1 University of Iowa Health Care; 2 University of Iowa

## Abstract

**Background:** Diverticulitis is a common cause of emergency department (ED) visits for abdominal pain. Although recent guidelines recommend selective antibiotic use for low-risk cases, antibiotics remain commonly prescribed. Facility-level variation in prescribing practices has been underreported. **Methods:** We conducted a retrospective cohort study (2016–2023) using the Veterans Health Administration (VHA) Corporate Data Warehouse. We identified ED visits for a first episode of diverticulitis and excluded patients admitted within 48 hours. Low-risk cases were defined using International Classification Disease-10 codes for diverticulitis without perforation or abscess, Charlson Comorbidity Index <2, white blood cell (WBC) count <15 x 10^3 /?L, and C-reactive protein <14 mg/dL. We applied a multivariable mixed-effects logistic regression model with random facility intercepts to assess variation in antibiotic prescribing across VHA facilities, adjusting for patient demographics, WBC count, and facility complexity level. Adjusted odds ratios (ORs) and 95% confidence intervals (CIs) were estimated to assess the associations between patient-level variables and antibiotic prescription. Facility-level variation was quantified using the median odds ratio (MOR). Bootstrap resampling (2,000 iterations) was used to estimate 95% CIs for the MOR. **Result:** Among 4,214 patients (median age, 51 years; 84.6% male) from 72 facilities with ?10 eligible encounters, 176 (4.2%) did not receive antibiotics and 4,038 (95.8%) received antibiotics at ED discharge. Compared with patients who received antibiotics, those not receiving antibioitcs were less often White (72.1% vs. 76.7%). Abnormal WBC count was associated with antibiotic prescribing in the multivariable logistic regression model (adjusted OR 2.15 [95% CI: 1.13-4.08], p=0.019; Table). Facility-specific adjusted ORs ranged from 0.26 (95% CI: 0.14-0.48) to 1.50 (95% CI: 0.60-3.79), indicating heterogeneity in prescribing practices across the VHA system (Figure). The MOR for the facility-level variation in antibiotic prescribing was 1.70 (95% bootstrap CI: 1.00-2.22), suggesting a median 70% difference in the odds of receiving antibiotics between two otherwise identical patients treated at randomly selected facilities. In one significantly low-prescribing facility, review of 63 providers caring for 83 patients showed that among 13 providers who saw low-risk diverticulitis on more than three occasions, 10 prescribed antibiotics in all encounters, 2 in 50-99% of encounters, and 1 in 33%, suggesting provider-level variation. **Conclusion:** In this nationwide VHA cohort of low-risk diverticulitis, antibiotic prescribing varied across facilities after accounting for patient characteristics and facility complexity. The MOR indicates significant facility-level variation in antibiotic prescribing across facilities, highlighting opportunities for targeted antibiotic stewardship and implementation strategies to improve guideline-concordant care.

**Figure:**
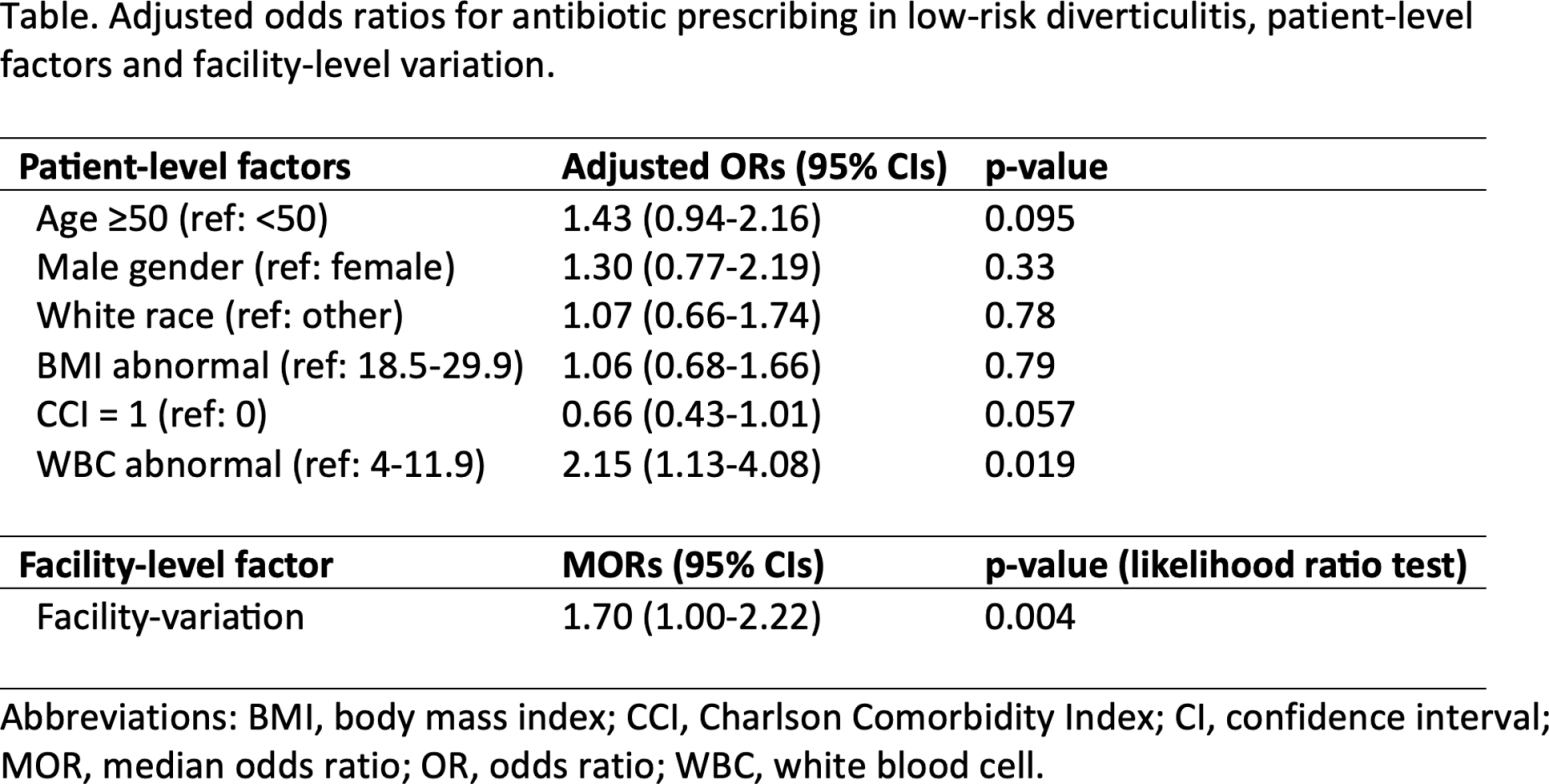

**Figure:**